# The Role of Feedback Training on Early Postoperative Recovery and Anxiety Scores in an Ambulatory Surgical Unit: A Secular Trend Study

**DOI:** 10.2478/jccm-2024-0036

**Published:** 2024-10-31

**Authors:** Alexander Dukhan, Teymur Yusupov, Naama Kabra, Tiberiu Ezri, Mona Boaz

**Affiliations:** Ambulatory Surgery Unit, Anesthesiology, Kaplan Medical Center, Rehovot, Israel; Tel Aviv University, Israel| Kaplan Medical Center; Ariel University, School of Health Sciences, Ariel, Israel

**Keywords:** feedback training, PACU, recovery score, performance assessment, secular trend, anesthesia

## Abstract

**Background:**

We used a ten-item postoperative quality of recovery score (QoR-10) to assess the perioperative quality of care in an in-hospital ambulatory surgical unit.

**Methods:**

In Phase 1 of this secular trend study (n=300 patients, 3-months duration), we collected QoR-10 scores and potential confounders, including type of anesthesia and surgery; co-morbidities; and anesthesia components of the Amsterdam scale-measured anxiety scores. Phase 2 was the one-month performance feedback learning phase in which modifiable variables identified in Phase 1 were translated to actionable steps, reinforcing the already existing routine of our department’s clinical practices, including pain, shivering and anxiety. The anesthesiology team was instructed and reminded of these steps using performance feedback methods. In Phase 3 (n=300 patients, 3-month duration) we evaluated the efficacy of this performance feedback instruction. QoR-10 scores were compared between Phase 1 and Phase 3.

**Results:**

Phase 1 identified three modifiable variables as targets for improvement: postoperative shivering; percentage of patients with numerical rating pain scale (NRS)<4; and preoperative anxiety from anesthesia scores. Compared to Phase 1, significantly fewer Phase 3 patients had severe shivering (2.3% vs. 7.3%, p = 0.023), and a greater percentage had NRS < 4 points (79% vs. 49.7%, p <0.001). The percentage of patients with a high anxiety score did not differ between phases. A direct association between anxiety score and QoR-10 score was not detected. The QoR-10 score (median (IQR)) was significantly higher in Phase 3 than Phase 1: 50 (49–50) vs. 49(49–50), p<0.001. In a multivariable logistic regression analysis, odds for a QoR-10 score of 49–50 were 1.92 higher in Phase 3 than Phase 1.

**Conclusion:**

Considering the study limitations, team feedback education contributed to improvement of the QoR-10 score, reduced the proportion of patients with severe shivering and increased the percentage of patients with low pain scores.

## Introduction

The quality of post-surgery recovery may reflect the caliber, type and duration of anesthesia [1], influenced by the rate and severity of immediate postoperative complications. More than 50% of the untoward incidents in the PACU (Post-Anesthesia Care Units) occur in ASA PS (American Society of Anesthesiologists Physical Status) 1 and 2 patients [2].

Besides comorbidities, health status, and type of surgery, postoperative patients’ outcome and satisfaction after ambulatory surgery were also affected by patient’s health literacy, age, and mental health status [3]. Shared decision-making and detailed preoperative and postoperative information may increase patient satisfaction [3]. Older patients reported better quality of recovery after surgery, but the reason for this is unclear [3].

Shivering may increase oxygen consumption leading to increased catecholamine release, which can be detrimental in elderly patients and in those with coronary heart disease and has been shown to amplify post-surgical pain and cause even more discomfort than nausea and vomiting [4,5].

Preoperative anxiety was found to be a predictor of morbidity and less consistently, mortality in patients aged > 70 years undergoing cardiac surgery [6,7].

Appropriate perioperative pain management, including the use of multimodal analgesia techniques improves postoperative recovery and enables early ambulation [8].

Closed claims cases [9], continuing medical education, team member evaluations and interactions [10] and patient questionnaires can contribute to the improvement of postoperative care quality. Performance feedback facilitates professional competence for physicians [11, 12]. Assessment of quality of recovery is a complex process; therefore, the use of a practical and comprehensive assessment tool is essential.

The Quality of Recovery from Surgery and Anesthesia 15-point score (QoR-15) is a simplified test for assessing the quality of postoperative recovery in adults [1,13]. This questionnaire was derived from a previously published 40-item QoR questionnaire [14]. The QoR-15 test of postoperative quality of care measurement after ambulatory surgery has also been evaluated [13]. In our ambulatory surgery unit, we developed a simplified, ten-item quality of recovery score (QoR-10), based on a patient data extraction form that records information from the time of PACU admission, until the time of PACU discharge, and evaluates early postoperative recovery. The QoR-10 was based on information acquired during the PACU stay only, preventing bias due to loss to follow-up after patient discharge home.

### Objectives and hypotheses

The present study aimed to examine the influence of anesthesia team feedback education on targeted outcomes including anxiety, pain and shivering. The research hypotheses were as follows: H1.1: Feedback education influences anxiety scores; H1.2: Feedback education influences pain scores; H1.3: Feedback education influences shivering; H1.4: QoR-10 differs between Phase 1 vs. Phase 3 of the study (see Methods section).

## Methods

This single-center, secular trend study was approved by the Institutional Ethics Committee, Kaplan Medical Center, Rehovot, Israel (study protocol number 0213-21KMC). The study was undertaken at the Kaplan Hospital Ambulatory Surgery Unit, Rehovot, Israel. Phase 1 was conducted between October 2022-December 2022; the second phase was carried out from January to February 2023; and the third phase was performed between February 2023 and April 2023.

### Study Population

Included in the study were all ASA PS I-III patients, aged 18–85 years, undergoing surgery under general anesthesia in our in-hospital ambulatory surgery unit. The first 300 consecutive patients were included in Phase 1, and, another 300 patients were studied in Phase 3. Excluded were patients aged less than 18 years or older than 85 years of age; pregnant; those suffering from known cognitive or psychiatric disorders; patients suffering from opiate overuse, or addiction to recreational drugs.

### Quality of Recovery Score: QoR-10

The 10-item Quality of Recovery Score (QoR-10) was modified from the QoR-15 [13] and validated (see later) by our department. The maximal score for each item was 5 accounting for a total maximal score of 50 for the ten items included in the.

The ten elements of our QoR-10 score were: the patient’s ability to speak easily, the patient did not experience depression, the patient communicated easily with surrounding people, he breathed easily, he reported no mental distress, he reported no nausea/vomiting, he reported no other complaints, he moved/walked easily, he reported no shivering and reported a pain score < 4.

Pain was measured using a numerical rating scale (NRS), a generic tool by which patients describe their pain numerically, where 0=no pain and 10=worst pain conceivable. A score > 3 points triggers analgesia intervention [15].

Intraoperative pain management was tailored to the type of surgery and patient’s characteristics and included the use of fentanyl or continuous use of remifentanil, pain adjuvants such as paracetamol and dexamethasone, and when indicated and appropriate, topical anesthesia, wound infiltration or regional blocks were added. Postoperative pain assessed with NRS was managed with either morphine or meperidine, along with intravenous paracetamol or dipyrone.

### Anxiety

Preoperative anxiety was measured using a modified (by our team – see reasoning later) Amsterdam Perioperative Anxiety and Information Scale [16]. This was performed by the attending anesthesiologist for a given patient. The anxiety from anesthesia and information about it score was not a part of the QoR-10 score, but it was recorded by the treating anesthesiologist, since preoperative anxiety could have a role in the quality of patient recovery.

The original Amsterdam Perioperative Anxiety and Information Scale (16) contains six variables, three related to anesthesia and three related to surgery:

1. I am worried about the anesthetic
2. The anesthetic is on my mind continually
3. I would like to know as much as possible about the anesthetic
4. I am worried about the procedure
5. The procedure is on my mind continually
6. I would like to know as much as possible about the anesthetic

Each variable receives from 1 (minimal anxiety) to 5 (extreme anxiety) points, accounting a total of 30 points (maximum anxiety and need for information from anesthesia and surgery). Since we could not have control over the surgeons’ preoperative education of patients, we measured only anxiety from and need for information about anesthesia, accounting a maximum anxiety from anesthesia points of 15. We considered as severe anxiety from anesthesia a score>10. This may also contain in it the patient’s need for more information about anesthesia, however for the sake of simplicity, we called it anxiety from anesthesia.

To reduce anxiety regarding anesthesia, anesthesiologists answered all patients questions regarding anesthesia in the preoperative clinic.

### Validity and reliability

#### Face validity

Does the score obviously measure quality of postoperative recovery? – The QoR-10 was based on a preexisting score but added clinical signs associated with successful postoperative recovery and altered others to make them more directly measurable.

#### Expert validity

Do experts (anesthesiologists) agree that the test measures what it is intended to measure? - To ensure expert validity, two attending anesthesiologists participating in our study, chose the variables that might have an effect on the QoR-10 score. The variables were taken from QoR-10 scores described previously in the literature. Additionally, we changed some of these variables, i.e. instead of classifying pain as moderate or severe as had been done in previous studies, we used a measurable, NRS scale for pain assessment. Additionally, we added three more variables, not included in previous quality of postoperative recovery scores: not feeling shivering (this may have a serious impact on the quality of the immediate postoperative recovery), ease of speaking; and not having other complaints. The two attending anesthesiologists analyzed all these components and agreed upon their inclusion in the score.

### Predictive validity

Does the score predict an outcome? - We measured one score to predict another one. QoR scores from Phase I were used to predict Phase III scores.

#### Reliability

Reliability was calculated using Cronbach’s alpha, the value of which was 0.6.

### Study Procedures

The study consisted of three phases. In Phase 1, data were collected from the records of 300 patients using the QoR-10. In Phase 2, modifiable variables in the QoR-10 were identified and targeted for change using performance feedback, during which anesthesiology team members were reminded to employ the routine clinical recommendations already applied in the department before this study, i.e. warming all patients, administering analgesics as needed and treating postoperative shivering as usual. Phase 3 included data collection from another 300 patients using the QoR-10.

### Feedback training

In Phase 2, the anesthesiology team participated in a one-month performance feedback education program to identify ways to improve the variables that received low scores on the QoR-10. Targeted measures included treating shivering, pain, and pre-operative anxiety.

All department anesthesiologists (n=7), who were specialist-attending physicians, were instructed to adopt the following practices: warm all patients as soon as they arrive in the OR and after surgery in the PACU; pay attention to treat intraoperative pain per department routine, depending on the procedure, patient age, comorbidities, and other factors; use regional blocks and local anesthesia per anesthesiologist’s clinical judgement and routine, in addition to general anesthesia; provide detailed explanations to patients regarding the process of anesthesia, as part of the pre-anesthesia assessment, in order to reduce preoperative anxiety related to anesthesia. We did not add special drugs or techniques during this educational phase, but rather emphasized to reinforce the use of known drugs and techniques.

During Phase 3, QoR-10 scores were recorded in another 300 patients undergoing surgery in our ambulatory surgical department during a 3-month data collection period. QoR-10 scores were compared between Phase 1 and Phase 3.

In addition to the QoR-10 items and anxiety scores, the following variables were recorded for each patient in Phase 1 and Phase 3: type of surgery; type of anesthesia; comorbidities; duration of anesthesia; duration of PACU stay, complications encountered and pre-planned and unplanned admission to the hospital for overnight stay after ambulatory surgery.

### Sample size

It was calculated that a sample size of 296 patients in each of the data collection phases (Phase 1 and Phase 3) was required to achieve a study power of 80%; the effect size was set at 0.06, assuming that the percentage of participants with QoR-10 scores 49–50 points would increase from 77% prior to the feedback education (Phase 1) to 85% after the feedback education (Phase 3). The two-tailed alpha was set at 0.05.

### Data Analysis

All analyses were carried out using SPSS.25 software (IBM, USA). Distributions of continuous variables were tested for normality using the Kolmogorov-Smirnov test and described as mean ± standard deviation or median (interquartile range [IQR]) as appropriate to variable distribution. Nominal variables were summarized in frequency tables. Ordinal variables were tested for normality using Shapiro–Wilk test and compared by the non-parametric Mann-Whitney-U test. Continuous variables were compared between the study groups using T test for independent samples. Categorical variables were compared using Pearson Chi-square test. Potential confounders were tested in univariable analyses of associations between them and QoR-10, anxiety, shivering and pain outcomes. Confounders were controlled for in logistic analyses. All tests were two-sided and considered significant at p-value<0.05.

The final analysis was done using multivariable logistic regression.

The primary outcomes of the present study are the differences between QoR-10 scores measured prior (Phase 1) vs. after (Phase 3) performance feedback learning implemented in Phase 2. Secondary outcomes include changes from Phase 1 to Phase 3 in each of the three modifiable variables identified in Phase 1: shivering, pain, and anxiety from anesthesia scores.

All the QoR-10 scores were calculated based on the nurses’ measurements, right before the patient discharge home or (if pre-planned) to hospital ward. This timing has the advantage of further treating specific problems if this was necessary, in order to fulfill the validated Modified Post Anesthetic Discharge Scoring System criteria [17].

## Results

Table 1 shows the patients and hospitalization characteristics.

**Table 1.** Patient and hospitalization characteristics, Phase 1 (n=300) vs. Phase 3 (n-300)

**Variable**		**Summary Measure**		**Significance by chi-square**
		**Phase 1**	**Phase 3**	
Age (median (IQR))		50 (36–65)	53(36–66)	0.734

Sex (n(%))	Female	121(40.3)	101(33.7)	0.091
	Male	179(59.7)	199(66.3)	

ASA (n(%))	1	146(48.7)	117(39)	0.053
	2	136(45.3)	168(56)	
	3	18(6)	15(5)	

Anesthesia type (n (%))	Balanced	9 (3.0)	17(5.7)	0.275
	TIVA	247 (82.3)	241(80.3)	
	TIVA + block	44 (14.7)	42(14.0)	

Preplanned hospitalization* (n (%))	No	201(67)	226(75.3)	0.024
	Yes	99(33.0)	74(24.7)	

Surgery Type	Orthopedics	75 (25.0)	75 (25.0)	1.000
	ENT	65 (21.7)	67(22.3)	0.844
	General surgery	58 (19.3)	66(22.0)	0.465
	Urology	33 (11.0)	57(19.0)	0.06
	Breast	20 (6.7)	2(0.7)	0.000
	Plastic	12 (4.0)	6(2.0)	0.151
	Vascular	10 (3.3)	10(3.3)	1.000
	Maxillo-facial	9(3.0)	9 (3.0)	1.000
	Eye	8(2.7)	6(2.0)	0.569

Comorbidities	Hypertension	116(38.7)	73(24.3)	0.777
	Diabetes mellitus	76(25.3)	13(11.0)	0.117
	Smoking	46(15.3)	28(9.3)	0.058
	Obesity	43(14.3)	41(13.7)	0.261
	Cardiac	32(10.7)	0(0.0)	0.000
	Asthma	14(4.7)	12(4.0)	0.832
	COPD	11(3.7)	6(2.0)	0,433
	Hypothyroidism	9(3.0)	0(0.0)	0.003
	Renal disease	9(3.0)	0(0.0)	0.004
	Chronic anxiety	8(2.7)	0(0.0)	0.014

Anesthesia time (median, IQR)		63(44–97)	60(37–100)	0.040

PACU time (median, IQR)**		90(60–120)	100(80–129)	0.000

* There were no unplanned hospitalizations;

** PACU=post anesthesia care unit

Table 2 shows the distribution of QoR-10 scores and of each item in the QoR-10 score as well as the anxiety from anesthesia scores in phases 1 and 3 of the study.

**Table 2.** Distribution of score = 5 (indicating best recovery) for each item in the QoR-10 score* and anxiety from anesthesia scores. Phase 1 (n=300) vs. Phase 3 (n-300)

**Measure**	**Percentage of population with score = 5 (maximum score for each item)**		**Significance by chi-square**
	**Phase 1**	**Phase 3**	
Spoke easily	99.0%	99.7%	0.512
Did not experience depression	99.0%	99.7%	0.570
Communicated easily with surrounding people	99.0%	100%	0.389
Breathed easily	98.0%	99%	0.221
Reported no mental distress	97.7%	97.3%	0.570
Reported no nausea/vomiting	96.3%	97.3%	0.731
Reported no other complaints	95.3%	94%	0.489
Moved/walked easily	93.3%	97.3%	0,146
Reported no shivering	92.7%	97.7%	0.023
NRS score < 4**	49.7%	79%	0.000
QoR-10 score (median, IQR)	49(49–50)	50(49–50)	<0.001

Anxiety from anesthesia	5.92(3.55)	5.78(3.14)	0.617
- Worry about anesthesia	11%	8.3%	0.495
- Thoughts about anesthesia	9%	7.7%	0.494
- Wanted to know more about anesthesia	5%	6.3%	0.560

* The maximum total score for the 10 items is 50;

** NRS=numerical rating scale

The median QoR-10 score in Phase 1 of the study was 49 out of 50, which is very high and suggests excellent recovery in most patients. The QoR-10 score in Phase 3 was even higher (Table 2).

In both phases, the QoR-10 scores were dichotomized into two categories based on QoR-10 score: score ≤ 48 vs. score > 48 (maximal scores). Demographic, medical history and anesthesia variables were compared by the dichotomized QoR-10 score. In both phases of the study, patients in the high score group were significantly older than patients in the low score group - 51 years (38–69) vs. 45 (30–59) in Phase 1 and 51 years (37–67) vs. 41 (31–50) in Phase 2). Anesthesia time was shorter in Phase 1, but duration of time in the PACU was significantly longer in Phase 3 (Table 1), mainly on account of the low QoR-10 score patients −144 min (90–150) in low score patients vs. 107 min (80–120) in high score patients.

Table 1 also shows that (due to surgical reasons), there were significantly more preplanned hospital admissions in phase 3 compared to phase 1, as well as less renal and cardiac diseases and less chronic anxiety.

The two items with the lowest proportion of participants with QoR-10 = 5 in Phase 1 were: reported no shivering (92.7%) and NRS pain score < 3 (49.7%). These two items significantly improved in Phase 3 (Table 2).

In addition to the QoR-10 score, in Phase 1, 31% patients had anxiety scores > 10. Almost half of all patients (49.3%) reported concern about anesthesia. A total of 37.3% patients wanted to know more about anesthesia. These anxiety results were not significantly improved in Phase 3 (Table 2)

We ran a logistic regression model with all cases from phase 1 and phase 3 together, with the dependent variable being the QoR-10 dichotomized up to 48 (category =0) versus 49–50 (category =1).

The independent variables were entered were:

0-female, 1-male, kind of anesthesia-TIVA-1, other-0, age, anesthesia time, PACU time, 0-no health problem. 1-health problem, general surgery 0-no, 1-yes, anxiety-score till 10, score >10.

QoR – in phase 3 was compared to QoR in phase 1.

As age increases, the chance of a score of 49–50 increases 1.03 times. As PACU time increases, the chance of having a lower score (48 or less) increased. Men have 1.76 higher chances of a score of 49–50 than women.

People with disease have 0.58 lower chances for high score 49–50. People without disease have 1.72 times more change of getting high score 49–50.

Type of surgery did not influence the score.

For Phase 3 chances for a QoR-10 score of 49–50 is 1.92 higher than for Phase 1.

Type of surgery and anesthesia anxiety score did not influence the QoR-10 score.

## Discussion

The present study identified shivering, pain and anesthesia anxiety secondary outcomes, and targeted them for improvement in an educational feedback training program for anesthesiology staff. After a month of this training, measures were repeated. Shivering and pain scores significantly improved. Scores that assess the quality of recovery from anesthesia, such as the Aldrete method [18] or modified post anesthetic discharge scoring system – MPADSS [17] do not assess the overall wellbeing of the patient during recovery. We added the NRS scale to more exactly evaluate pain [15].

As emphasized in the Introduction section, shivering, anxiety and pain might have a negative influence on the postoperative outcomes [4,5,6,7,8].

Shivering was a modifiable outcome successfully targeted in the present study.

Postoperative pain was frequently observed in Phase 1 of our study. Thus, during Phase 2, anesthesiologists were instructed to pay attention to intraoperative and postoperative pain management, by using departmentally routine analgesia. Indeed, after this guidance in Phase 2, we observed a significant increase in the percentage of patients with NRS<**4** in Phase 3 (79% vs. 49.7%, p<0.001).

In the present study, anxiety score did not significantly decline from Phase 1 to Phase 3. It is possible that this reflects inadequate surgeon’s explanation to the patient of the surgical processes. However, as shown in Table 2, the overall anxiety from anesthesia scores in both phases were relatively lor (<6).

We cannot explain why older patients had higher QoR-10 scores. Others have also had difficulty explaining this [3]. The connection between longer PACU stay and lower QoR-10 score is logical reflecting the need for longer PACU treatment in patients with lower QoR-10.

In regard to a possible confounding influence of seasonal variation during the study period (Phase 1 was conducted between October 2022–December 2022; the second phase was carried out from January to February 2023; and the third phase was performed between February 2023 and April 2023), this is a rainy period in Israel so we do not believe it could have any influence on our study findings. Also, other timing confounders can be excluded since our day surgery unit works constantly five times per week from 7 AM to 4 PM.

The present study shows that feedback training can contribute to improvement of postoperative quality of care, suggesting that staff education can be an effective intervention for improving patient outcomes [19]. This is mainly reflected by a significant improvement of our primary outcome, the QoR-10 score in phase 3 of the study. Also, our secondary outcomes shivering and NRS significantly improved.

While our study found that feedback training was associated with improvements in postoperative shivering and pain, others, using another scoring system have also shown improvements in one-month complication rates after elective surgery [20].

### Limitations of the study

The main limitation of the study (but at the same time emphasizing its originality) is the lack of follow up time after the PACU state. A longer follow-up duration would eventually identify even rare adverse events and would also give an opportunity to estimate patient satisfaction with their perioperative management. Additionally, we did not find a change in the anxiety score between phase 3 and phase 1, possibly attributable to variability of explanations among anesthesiologists in the preoperative clinic, and to the lack of data regarding anxiety from surgery. This was caused by the lack of our control on surgeons’ education in phase 2 of the study.

Findings of the present study indicate that feedback education is a feasible tool for identifying actionable clinical goals, targeting them for improvement. This method may have a role in the continuing education of anesthesiologists. Additionally, the QoR-10 score can be used to assess anesthesia team performance.

However, considering the limitations depicted above, further studies are necessary to reconfirm the routine clinical usefulness of QoR-10.

## References

[1] Stark PA, Myles PS, Burke JA (2013). Development and psychometric evaluation of a postoperative quality of recovery score. The QoR-15. Anesthesiology.

[2] Kluger MT, Bullock FM (2002). Recovery room incidents: a review of 419 reports from the Anaesthetic Incident Monitoring Study (AIMS). Anaesthesia.

[3] Jaensson M, Karuna Dahlberg K, Nilsson U (2019). Factors influencing day surgery patients’ quality of postoperative recovery and satisfaction with recovery: a narrative review. Periop Med (Lond).

[4] Bilotta F, Pietropaoli P, La Rosa I, Spinelli F, Rosa G (2001). Effects of shivering prevention on haemodynamic and metabolic demands in hypothermic postoperative neurosurgical patients. Anaesthesia.

[5] Alfonsi P (2001). Postanaesthetic shivering: epidemiology, pathophysiology, and approaches to prevention and management. Drugs.

[6] Williams JB, Alexander KP, Morin JF (2013). Preoperative anxiety as a predictor of mortality and major morbidity in patients aged > 70 years undergoing cardiac surgery. Am. J. Cardiol..

[7] Kassahun WT, Mehdorn M, Wagner CT, Babel J, Danker H, Gockel I (2022). The effect of preoperative patient-reported anxiety on morbidity and mortality outcomes in patients undergoing major general surgery. Sci Rep..

[8] Joshi GP, Kehlet H, on behalf of the PROSPECT Working Group (2017). Guidelines for perioperative pain management: need for re-evaluation. Br J Anaesth.

[9] Kellner DB, Urman RD, Greenberg P, Brovman EY (2018). Analysis of adverse outcomes in the post-anesthesia care unit based on anesthesia liability data. J Clin Anesth.

[10] O‘Brien MK, Dexter F, Kreiter CD, Slater-Scott C, Hindman BJ (2019). Nurse anesthetists’ evaluations of anesthesiologists’ operating room performance are sensitive to anesthesiologists’ years of postgraduate practice. J Clin Anesth.

[11] Kaye AD, Okanlawon OJ, Urman RD (2014). Clinical performance feedback and quality improvement opportunities for perioperative physicians. Advances in Medical Education and Practice.

[12] D’Lima D, Arnold G, Brett SJ, Bottle A, Smith A, Blenn J (2017). Continuous monitoring and feedback of quality of recovery indicators for anaesthetists: a qualitative investigation of reported effects on professional behaviour. Br J Anaesth.

[13] Chazapis M, Walker EMK, Rooms MA, Kamming D, Moonesinghe SR (2016). Measuring quality of recovery-15 after day case surgery. Br J Anaesth.

[14] Myles PS, Weitkamp B, Jones BK, Melick J, Hensen S (2000). Validity and reliability of a postoperative quality of recovery score: the QoR-40. Br J Anaesth.

[15] Gerbershagen HJ, Rothaug J, Kalkman CJ, Meissner W (2011). Determination of moderate-to-severe postoperative pain on the numeric rating scale: a cut-off point analysis applying four different methods. Br J Anaesth.

[16] Moerman N, van Dam F, Muller MJ, Oosting H (1996). The Amsterdam preoperative anxiety and information scale (APAIS). Anesth Analg.

[17] Palumbo P, Tellan G, Perotti B, Pacilè MA, Vietri F, Illuminati G (2013). Modified PADSS (Post Anaesthetic Discharge Scoring System) for monitoring outpatient discharge. Ann Ital Chir.

[18] Aldrete J. A, Kroulik D. (1970). A Postanesthetic Recovery Score. Anesth Analg.

[19] Weller J, Gotian R (2023). Evolution of the feedback conversation in anaesthesia education: a narrative review. Br J Anaesth.

[20] Campfort M, Cayla C, Lasocki S, Rineau E, Léger M (2022). Early quality of recovery according to QoR-15 score is associated with one-month postoperative complications after elective surgery. J Clin Anesth.

